# A case of upper gastrointestinal bleeding due to pancreatic pseudoaneurysm rupture

**DOI:** 10.1002/kjm2.12810

**Published:** 2024-02-16

**Authors:** Hui‐Qi Siew, Ming‐Lun Yeh

**Affiliations:** ^1^ School of Medicine, College of Medicine Kaohsiung Medical University Kaohsiung Taiwan; ^2^ Division of Hepatobiliary, Department of Internal Medicine Kaohsiung Medical University Hospital Kaohsiung Taiwan

Pseudoaneurysm was an uncommon complication of acute pancreatitis, which was occurred when an erosion of a peripancreatic or pancreatic artery into a pseudocyst.^1^ Pancreatic pseudoaneurysm might rupture or form a fistula into the adjacent hollow organs, causing a life‐threatening bleeding. Herein, we reported a case of severe upper gastrointestinal (GI) bleeding due to pancreatic pseudoaneurysm ruptured in the stomach.

The patient was a 50‐year‐old male with a history of alcoholism related recurrent pancreatitis. The most recent episode of his acute pancreatitis was 3 months ago. At that episode, pancreatic head pseudocyst compressing the pancreatic duct causing an intolerable abdominal pain were found. An endoscopic ultrasound guide cystogastrostomy with a plastic stent to relief pseudocyst was performed. Following ultrasound showed resolution of pancreatic pseudocyst thereafter. This time, he visited the emergency department with the chief complaint of tarry stool for 2 weeks. Severe epigastric pain was also noted over the past 3 days, along with hematemesis. Upon admission, his vital signs were stable and the esophagogastroduodenoscopy revealed an active duodenal ulcer with much fresh blood in the stomach. Intravenous proton pump inhibitor was immediately prescribed. However, the patient experienced a significant episode of hematemesis at midnight, resulting in the vital sign change of tachycardia and hypotension. After the emergent managements to keep vital sign stable, computed tomography (CT) scan was done, and revealed a pseudoaneurysm originating from the gastroduodenal artery (Figure 1A), accompanied by hematoma surrounding the duodenum, pancreatic head, and extending into the gastric lumen. The emergent angiography was performed with subsequent gelfoam and coils embolization through the gastroduodenal artery (Figure 1B,C). After procedure, his hemoglobin levels stabilized. A follow‐up CT scan at 9 weeks post‐treatment showed successful coil embolization of the gastroduodenal artery pseudoaneurysm and a reduction in the size of the cyst or cystic neoplasm at the pancreatic head (Figure 1D).

**FIGURE 1:**
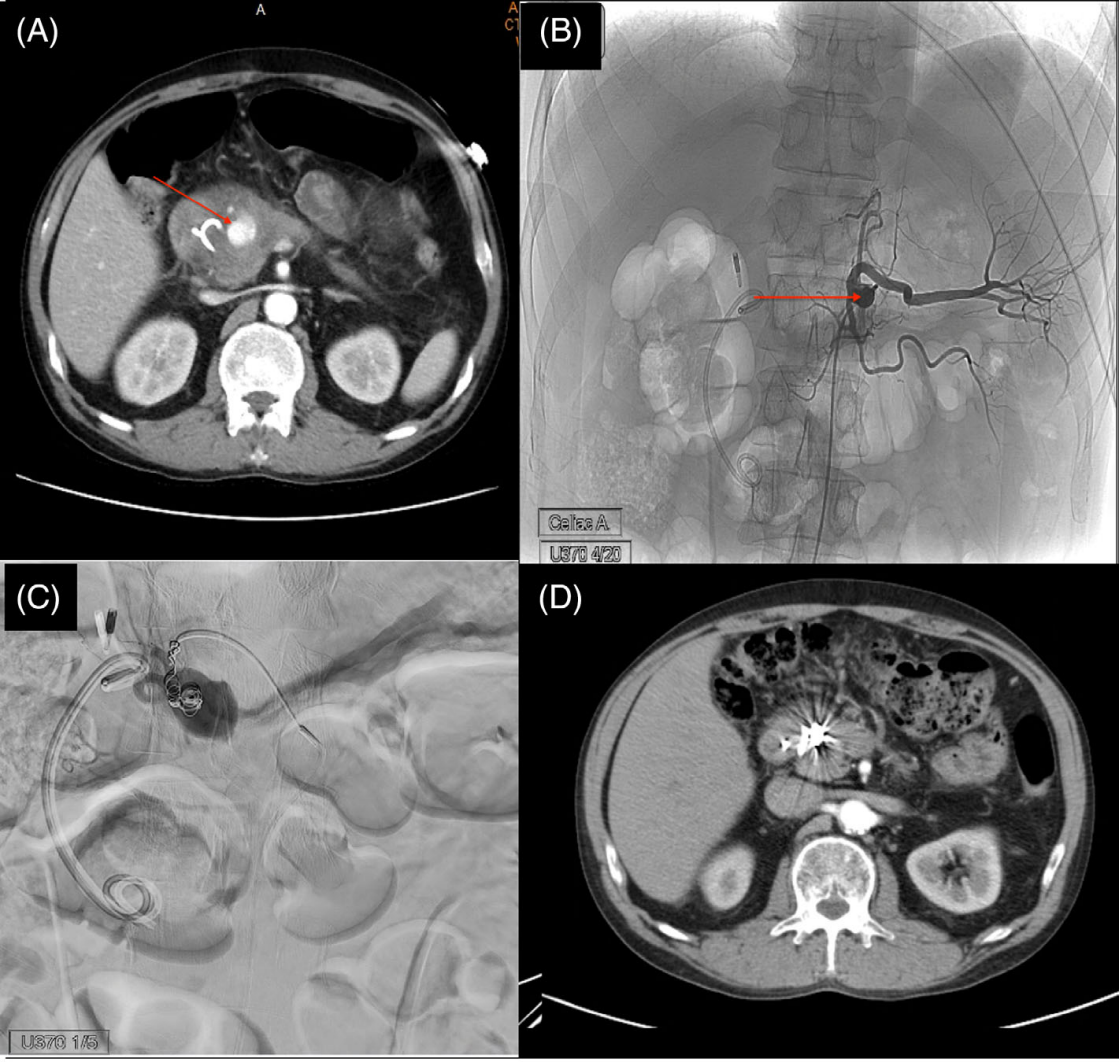
Contrast enhanced abdominal computed tomography (CT) scan showed pseudoaneurysm (arrow) originating from the gastroduodenal artery (A). Angiogram showed a compatible result with CT scan (B). Angiographic embolization with combination of coils and gelfoam was introduced to gastroduodenal artery (C). Followed CT scan showed successful coil embolization of the pseudoaneurysm and reduction in the size of the pseudocyst at pancreatic head (D).

Pancreatic pseudoaneurysm may be developed through two primary mechanisms. First, proteolytic enzymes within pseudocysts could self‐digest adjacent artery walls, leading to aneurysm formation. Second, pseudocysts may persistently compress nearby arteries, causing ischemic vessel damage.^2^ The splenic artery was most frequently affected (30%~50%). Following this, the gastroduodenal and pancreaticoduodenal arteries each had a 10% involvement rate. The mortality rate could reach 90% in untreated patients but dropped to ~12.5% with treatment.^3^ Pancreatic pseudoaneurysm should be suspected in patients with (1) acute or chronic pancreatitis, especially those with active alcohol consumption; (2) unexplained GI bleeding without an identifiable source; (3) GI bleeding accompanied by profound hypovolemic shock.^4^ CT angiography was the gold standard for diagnosis. Angiography confirmed the diagnosis, localizes the arterial source, and facilitates appropriate treatment through embolization.^5^ Other treatment options included urgent transcystic ligation with external drainage, and pancreatic resection. The choice between surgery and angiographic embolization depends on the patient's clinical presentation. Hemodynamically stable patients should receive angiographic embolization as initial treatment, while surgery is reserved for hemodynamically unstable patients. Follow‐up is essential, as these patients may experience pancreatic insufficiency or develop new pseudocysts.

## CONFLICT OF INTEREST STATEMENT

All authors declare no conflict of interest.

## References

[1] Hoilat GJ , Mathew G , Ahmad H . Pancreatic Pseudoaneurysm. Treasure Island, FL: StatPearls; 2023. 28613687

[2] Gurala D , Polavarapu AD , Idiculla PS , Daoud M , Gumaste V . Pancreatic pseudoaneurysm from a gastroduodenal artery. Case Rep Gastroenterol. 2019;13(3):450–455. 31762734 10.1159/000503895 PMC6873056

[3] Mallick B , Malik S , Gupta P , Gorsi U , Kochhar S , Gupta V , et al. Arterial pseudoaneurysms in acute and chronic pancreatitis: clinical profile and outcome. JGH Open. 2018;3(2):126–132. 31061887 10.1002/jgh3.12116 PMC6487818

[4] Chiang KC , Chen TH , Hsu JT . Management of chronic pancreatitis complicated with a bleeding pseudoaneurysm. World J Gastroenterol. 2014;20(43):16132–16137. 25473165 10.3748/wjg.v20.i43.16132 PMC4239499

[5] Gupta V , Irrinki S , Sakaray YR , Moond V , Yadav TD , Kochhar R , et al. Treatment strategies for bleeding from gastroduodenal artery pseudoaneurysms complicating the course of chronic pancreatitis—a case series of 10 patients. Indian J Gastroenterol. 2018;37(5):457–463. 30374751 10.1007/s12664-018-0897-y

